# Natural Product Alantolactone Targeting AKR1C1 Suppresses Cell Proliferation and Metastasis in Non-Small-Cell Lung Cancer

**DOI:** 10.3389/fphar.2022.847906

**Published:** 2022-03-15

**Authors:** Zhiwen Fu, Shijun Li, Jinmei Liu, Cong Zhang, Chen Jian, Lulu Wang, Yu Zhang, Chen Shi

**Affiliations:** ^1^ Department of Pharmacy, Union Hospital, Tongji Medical College, Huazhong University of Science and Technology, Wuhan, China; ^2^ Hubei Province Clinical Research Center for Precision Medicine for Critical Illness, Wuhan, China

**Keywords:** alantolactone, AKR1C1, anticancer, target identification, non-small-cell lung cancer

## Abstract

Non-small-cell lung cancer (NSCLC) is one of the leading causes of cancer-related deaths, characterized by high invasion and metastasis. Aldo-keto reductase family 1 member C1 (AKR1C1) plays an important role in cancer cell proliferation and metastasis, and has gained attention as an anticancer drug target. Here, we report that the natural sesquiterpene lactone alantolactone (ALA) was shown to bind directly to AKR1C1 through the Proteome Integral Solubility Alteration (PISA) analysis, a label-free target identification approach based on thermal proteome profiling. Acting as a specific inhibitor of AKR1C1, ALA selectively inhibits the activity of AKR1C1 and ALA treatment in human non-small-cell lung cancer (NSCLC) cell results in a reduction in cell proliferation and metastasis, inhibition of AKR1C1 expression, and deactivation of STAT3. Moreover, ALA inhibited tumor growth *in vivo*, and the inhibition of AKR1C1 and STAT3 activation were also found in the murine xenograft model. Collectively, our work not only gives mechanistic insights to explain the bioactivity of ALA in anticancer but also provides opportunities of developing novel sesquiterpene lactone-based AKR1C1 inhibitors for the treatment of NSCLC.

## Introduction

Aldo-keto reductase family 1 member C1 (AKR1C1), also known as 20α-hydroxysteroid dehydrogenase, is a member of the human aldo-keto reductase family (Mindnich and Penning, 2009). It catalyzes the conversion of aldehydes and ketones to their corresponding alcohols using NADH or NADPH as cofactors, which plays a regulatory role in a wide range of cellular processes, such as cell proliferation, metabolism, apoptosis, and ferroptosis (Zeng et al., 2017; Jiang et al., 2018; Huang et al., 2021). An increasing body of evidence reveals that AKR1C1 shows a higher expression in a wide variety of cancers, including lung cancer, gastric cancer, and cervical cancer, than normal corresponding tissues (Hong et al., 2018). Overexpression of AKR1C1 has been found to be involved in cancer initiation and progression, and it could contribute to proliferation and cell-death evasion in cancer cells (Matsumoto et al., 2016). In addition, AKR1C1 promotes cancer metastasis and clinically is correlated with poor prognosis (Hong et al., 2018). Moreover, AKR1C1 overexpression correlates with drug resistance in some cancer cell lines to chemotherapy *in vitro* (Wang et al., 2007; Bortolozzi et al., 2018; Chang et al., 2019). All these results suggest that AKR1C1 plays a complex role in carcinogenesis and represents an important target for cancer therapy.

Several AKR1C1-specific inhibitors have been identified, including steroidal and non-steroidal compounds, which interact with AKR1C1 as its substrates (Brožič et al., 2006; El-Kabbani et al., 2011). Of note, 3-bromo-5-phenylsalicylic acid, one of the salicylic acid derivatives, is a highly potent AKR1C1 inhibitor with Ki values in the nanomolar concentration, but little success has been applied in clinical trials (El-Kabbani et al., 2010). Currently, fewer natural compounds have been reported to show a specific inhibitory effect on AKR1C1 by direct binding to AKR1C1. Therefore, it is of substantial interest to explore more compounds that directly bind to AKR1C1 to suppress cell proliferation and metastasis for effective cancer therapy.

*Inula helenium* L. is one of the fundamental medicinal herbs that have long been used to treat diseases in traditional Chinese medicine, and its raw extracts have been reported to have antioxidant and anticancer activities (Koc et al., 2018; Yan et al., 2020). In particular, one of its major sesquiterpene lactone components, alantolactone (ALA), was shown to induce cell apoptosis and inhibit invasion and migration in non-small-cell lung cancer (NSCLC) (Maryam et al., 2017). The mechanism of action of ALA for inhibiting the invasion and migration of NSCLC is complex, and the exact mechanism has not been fully characterized. Existing pharmacological studies showed that ALA exerted antitumor activity mainly by inhibiting the phosphorylation of signal transducer and activator of transcription 3 (STAT3) and affecting various key signal molecules involved in cancer progression (Chun et al., 2015). For example, ALA could increase the level of reactive oxygen species (ROS) in cells and cause mitochondrial damage by inhibiting glutathione reductase (GR) and thioredoxin reductase (TrxR) (Zhang et al., 2016). In addition, ALA also passes eIF2 α to cause ER stress, inhibit autophagy, and finally induce apoptosis (He et al., 2018; Yin et al., 2019). However, the direct target of ALA remains unknown so far.

In the present work, we implemented Proteome Integral Solubility Alteration (PISA) analysis (Gaetani et al., 2019), a label-free chemical proteomic approach, to investigate the changes in protein thermal stability across the proteome, and identified AKR1C1 as one of the direct targets of ALA. Acting as a specific inhibitor of AKR1C1, we found ALA selectively inhibited the activity of AKR1C1 and inhibited the growth of NSCLC cells both *in vitro* and *in vivo*. Our work reports the discovery of a natural inhibitor of AKR1C1 for the treatment of NSCLC. The results not only give mechanistic insights to explain the bioactivity of ALA in anticancer but also provide opportunities of developing novel sesquiterpene lactone-based AKR1C1 inhibitors for pharmacological treatment of NSCLC.

## Materials and Methods

### Reagents

ALA (Cat# SML0415-25MG), (S)-(+)-α-Tetralol (Cat# 87649), and β-NADH phosphate disodium salt (Cat# 50020) were purchased from Sigma-Aldrich (MO, United States). Fresh ALA was dissolved in DMSO for each cell experiment (10 mM stock solution). Primary antibodies against GAPDH (Cat# ab181602), p-STAT3 (Ser727, Cat# ab32143), STAT3 (Cat# ab109085), and AKR1C1 (Cat# ab192785) were purchased from Abcam (Boston, MA, United States). The TMT10plex Isobaric Mass Tag Labelling Reagents (Cat# 90113) were obtained from Thermo Fisher Scientific (Boston, MA, United States). The Dual Luciferase Reporter Gene Assay Kit (Cat# RG028) was purchased from Beyotime (Shanghai, China). Cell Counting Kit 8 (CCK-8, Cat# ab228554) was obtained from Abcam (Boston, MA, United States).

### Cell Lines and Cultures

NCI-H460 cells were obtained from the American Type Culture Collection (ATCC, Manassas, VA, United States). The RPMI-1640 medium (Gibco, ThermoFisher Scientific) supplemented with 10% fetal bovine serum (Gibco, ThermoFisher Scientific) and 100 U/ml penicillin–streptomycin (Gibco, ThermoFisher Scientific) was used for cell culture. Cells were placed into an incubator with 5% CO_2_ and the temperature was set at 37°C. For subculturing cells, 0.05% trypsin-EDTA (Gibco) was used for detaching, and a passage ratio of 1:3 was used.

### Cell Viability Assays

The cell viability assay was performed using a Cell Counting Kit 8 (CCK-8) assay. Briefly, 5 × 10^3^ NCI-H460 cells were seeded in the 96-well flat bottom plate and allowed to attach overnight. After a specific time (24, 48, or 72 h) of treatment with varying concentrations of ALA, 10 μl of CCK-8 solution was added into each well and incubated for 2 h at 37°C. The absorbance at 460 nm was then directly measured by a microplate reader. The cell viability without treatment with ALA was normalized at 100%.

### Apoptosis Analysis

The apoptosis rate of cells was determined by flow cytometry using an Annexin V-FITC Apoptosis Detection Kit (APOAF-50TST, sigma) according to the manufacturer’s manual. Briefly, NCI-H460 cells were treated with the indicated concentrations of ALA for 48 h, and then harvested, washed with PBS, and centrifuged at 700 g for 3 min. Approximately 1 × 10^6^ cells were collected for staining and resuspended in 1 ml of binding buffer. Then, 5 µl of Annexin V-FITC conjugate solution (final concentration 2 µg/ml) and 10 µl of PI staining solution (final concentration 20 µg/ml) were added to the cell suspension. After 10 min of incubation at room temperature in the dark, the fluorescence was determined by the BD FACS CantoII Analyzer (BD Biosciences, CA, United States).

### Cell Wound Healing Assay and Transwell Invasion Assay

The cell wound healing assay was used to characterize the cell migration ability in the present study. Briefly, the NCI-H460 cells were seeded in the ibidi Culture-inserts 2 Well (Martinsried, Germany) at a density of 3 × 10^4^ cells per well. After allowing the cells to attach overnight, the Culture-inserts 2 Well was gently removed by using sterile tweezers and the non-attached cells were washed by PBS; then the remaining cells were continued to be cultured in the complete growth medium and treated with different concentrations of ALA or DMSO as vehicle control for 24 h. The formed cell-free gaps were, respectively, photographed at 0 and 24 h on each plate. Each condition was performed in triplicate.

The Transwell invasion assay was performed to study the cell invasion ability of NCI-H460 cells. Briefly, after 24 h of treatment with different concentrations of ALA (3, 10, and 30 μM), about 3 × 10^4^ NCI-H460 cells were suspended and re-seeded on the Transwell insert (upper chambers were pre-coated with ECM gel) in RPMI-1640 containing 1% FBS, whereas the lower chambers were loaded with RPMI-1640 containing 10% FBS as an attractant. After an incubation at 37°C for 30 h, the insert was taken out gently and the cells on the upper side of the insert membrane were removed with a cotton swab, while the invasive cells on the lower side of the insert membrane were fixed with 5% glutaraldehyde for 10 min, followed by staining with 0.1% crystal violet (dissolved in 2% ethanol) and counted under a microscope.

### Animals

All animal experiments in this project were approved by the Institutional Animal Care and Use Committee of Tongji Medical College, Huazhong University of Science and Technology. Eighteen six-week-old female BALB/c nude mice were purchased from Beijing Charles River Laboratories (Beijing, China). The mice were maintained in a pathogen-free environment (23 ± 2°C, 55 ± 5%) on a 12-h light/12-h dark cycle with food and water supplied *ad libitum* throughout the experimental period.

In the present work, the *in vivo* anticancer efficacy of ALA was evaluated using the human tumor xenograft model, in which human cancer cells were inoculated into immune-deficient mice. Briefly, 18 8-week-old (16–18 g) BALB/c nude mice were randomly divided into three groups, and both received subcutaneous injection of NCI-H460 cells (2 million cells in 0.1 ml PBS) at its right flank of mice and tumor became palpable after 3 days of injection. Mice received intravenous (*i.v.*) injections of ALA or vehicle control (5% DMSO and 0.05% Tween 80 in normal saline) every 2 days for 21 days. The length and width of tumor were measured by Vernier caliper, and the body weight was monitored every 2 days. At the end of the experiment, the mice were sacrificed and decapitated. The tumor tissues were collected to weight and freeze in liquid nitrogen for further immunoblot analysis. Tumor size and body weight were recorded before the drug injection every time, and tumor volume (V) was calculated by the formula V = LW^2^/2, where L is the tumor length and W is the tumor width. Data in each group were shown as the mean ± standard deviation.

### Immunoblotting

Cells were induced by different concentrations of ALA or varying treatment times, and then cells were harvested and washed twice in prechilled PBS. RIPA lysis buffer supplemented with the protease inhibitors (cOmplete, Mini, EDTA-free tablets, Roche) and phosphatase inhibitors (cOmplete™ ULTRA Tablets, Roche) was used for the extraction of protein and allowed for an incubation of 10 min on the ice. By centrifugation at 20,000 *g* for 10 min, the clear supernatant of the protein solution was obtained and transferred to the new Eppendorf tubes. The protein concentration was then measured by the BCA Protein Assay Kit (Pierce^TM^, ThermoFisher). An equal amount of protein (usually 30 µg) was aliquoted and mixed with the ×5 gel loading dye followed by the denaturation at 90°C for 5 min. The prepared protein sample was loaded onto the gel and separated by the sodium dodecyl sulfate-polyacrylamide gel electrophoresis (SDS-PAGE) using a constant voltage of 100 V for 2 h and then transferred to a polyvinylidene difluoride (PVDF) membrane (Amersham™, GE) by the Trans-Blot Turbo Transfer System (Bio-Rad). Afterward, the membranes were blocked with 5% BSA in Tris-buffered saline containing 0.1% Tween 20 (TBST) to reduce the non-specific binding at room temperature for 2 h. Subsequently, the membrane was incubated with the desired primary antibodies (at an appropriate dilution) at 4°C. After overnight incubation and three 10-min TBST, the membranes were incubated with corresponding HRP-conjugated secondary antibodies for 1 h at room temperature. The immunolabeling proteins on the membrane were observed on the Azure Biosystem C-200 after the development of an enhanced chemiluminescent (ECL) solution. The results were analyzed and processed using the ImageJ software (1.50i, National Institutes of Health, United States, http://imagej.nih.gov/ij).

### Proteome Integral Solubility Alteration Assay

NCI-H460 cells (5 × 10^6^) were seeded into 10 cm culture dishes and allowed for attachment overnight. The attached cells were treated in biological triplicates with DMSO (0.5%) and ALA (30 µM), respectively, for 2 h. Then, cells were harvested and washed twice with prechilled PBS, followed by a re-suspension using 500 µl of PBS. The cell suspensions were then aliquoted to ten PCR tubes and were heated at ten different temperatures (48, 49.2, 50.4, 51.6, 52.8, 54.0, 55.2, 56.4, 57.6, and 58.8°C), respectively, for 3 min followed by cooling for another 3 min. The cells were then lysed by three cycles of freeze-thaw using liquid nitrogen. The 10 aliquots from the same group were then combined into one sample, and the soluble protein fractions were collected by centrifugation at 100,000 *g* for 20 min at 4°C and transferred into new ultracentrifuge micro-tubes. The protein concentrations were measured using the BCA Protein Assay Kit (Pierce^TM^, ThermoFisher). An aliquot of an equal amount of protein from each group was then subjected to reduction and alkylation using dithiothreitol solution (DTT, final concentration 20 mM) and iodoacetamide solution (IAA, final concentration 50 mM), respectively. The protein samples were then precipitated using the methanol–chloroform mixture solution (methanol: chloroform = 4:1, *v/v*), and the obtained protein pellet was air-dried for 5 min at room temperature followed by re-dissolving with 8M urea buffer (dissolved in 20 mM HEPES buffer, pH 8.5, freshly prepared). By a dilution of the urea concentration to 4 M by the 20 mM HEPES buffer, the protein was digested by the Lys-C Protease (Pierce^TM^, MS grade, 90051, ThermoFisher) in a ratio of 1:50 (*w/w*) and incubated at room temperature overnight. The mixture was further digested by the trypsin protease (Pierce^TM^, MS grade, 90057, ThermoFisher) in a ratio of 1:75 (*w/w*) for 6 h at 37°C after diluting the urea concentration to 1 M. The acetonitrile was added to digested protein samples to a final concentration of 20% for the TMT labeling.

### TMT-Labeling

The TMT-10plex^TM^ Isobaric Label Reagent Set (0.8 mg, ThermoFisher, 90110) was used to label the above samples. The TMT reagent was dissolved in 20 µl super-dried acetonitrile solution and added to each sample as indicated, and the mixture was incubated at room temperature for 2 h. After quenching the reaction with 0.5% hydroxylamine, 15 samples were combined into one tube, and acetonitrile from the mixture solution was removed by the SpeedVac Vacuum Concentrators (ThermoScientific). Next, after the acidification of the peptide mixture to pH 2–3 using trifluoroacetic acid (TFA), the desalting process was carried out by the Sep-Pak C18 Vac Cartridge (1 cc, 50 mg, WAT054955, Waters) along with the Extraction Manifold Vacuum System (Waters) according to the manufacturer’s manual. The detailed desalting steps were as follows: the column was pre-washed with 450 µl of 100% methanol and equilibrated with 450 µl of 50% acetonitrile and 900 µl of 0.1% TFA. The peptide samples were loaded onto the columns followed by washing the salts twice with 450 µl of 5% acetonitrile in 0.1% formic acid. The peptides were eluted and collected in new Eppendorf tubes with 450 µl of 50% acetonitrile in 0.1% formic acid and 300 µl of 80% acetonitrile in 0.1% formic acid. The eluted peptide sample was submitted to SpeedVac vacuum concentrators until the samples were entirely dried. The dried sample was stored at −20°C before fractionation.

### Mass Spectrometry Analysis and Protein Identification

All peptides were re-dissolved using 10 µl of 0.1% formic acid and submitted for the LC-MS analysis. The analysis was carried out on an Easy-nLC^TM^ 1200 System (LC140, Thermo Scientific) in tandem with an Orbitrap Fusion^TM^ Tribrid^TM^ Mass Spectrometry (IQLAAEGAAPFADBMBCX, Thermo Scientific). The peptide separation was performed using the PepMap^TM^ RSLC C18 column (2 µm, 100 Å, 75 µm × 50 cm, Thermo Scientific, 10889337) with a binary solvent system consisting of 0.1% formic acid in water (Solvent A) and 0.1% formic acid in acetonitrile (Solvent B). A 240-min gradient with 150 µl/min of the flow rate was applied to the elution of mixture peptides: 0–20 min, 7–12% Solvent B; 20–220 min, 12–28% Solvent B; 220–230 min, 28–45% Solvent B; 230–232 min, 45–100% Solvent B; 232–240 min, 100% Solvent B.

The mass spectra were acquired using the positive ion scan mode with a spray voltage of 2,300 V. A data-dependent method was applied for the data acquisition. The Orbitrap full scan resolution was set at 120,000 with a scan range from 400 to 1,600 m*/z*. The precursor ions with charges ranging from 2 to 7 and intensities of over 5 × 10^4^ were selected for MS/MS analysis. The data-dependent MS/MS scan was performed by the higher-energy collision dissociation (HCD) methods with a 38 normalized collision energy value (NCE). The isolation window was set as 1.6 m/z and the first mass was started at 100 m/z. The data of MS^2^ scan were recorded with the resolution of 50,000.

For protein identification, the obtained raw data file was submitted to MaxQuant (version 1.6.10.43) for searching, identification, and quantification. The Fasta file of the Uniprot human proteome (29 April 2019, version UP000005640, 18,596 gene counts) was used to match the MS/MS sequences by using Andromeda as the search engine. TMT-10 quantification of peptide and protein abundances was selected. The carbamidomethylation at the cysteine terminal was used as a fixed modification, while oxidation at methionine was used as a variable modification for both identification and quantification. Trypsin/P was selected for the enzyme specificity with a maximum of two missed cleavages allowed. The protein identification false discovery rate (FDR) was filtered as 0.01 at both the protein and peptide level. The other parameters for searching were used as the default recommended settings.

### Cellular Thermal Shift Assay and Isothermal Dose-Response Fingerprinting Experiment

To prepare the samples for the CETSA analysis, NCI-H460 cells (5 × 10^6^) were seeded onto 10 cm culture dishes and allowed for overnight attachment. On the following day, cells were treated in biological duplicates with DMSO (0.5%) and ALA (30 µM), respectively, for 2 h. The cells were then harvested and washed twice with prechilled PBS, followed by a re-suspension using 500 µl of PBS. The cell suspensions were then aliquoted to ten PCR tubes and were heated at ten different temperatures (37, 41, 44, 47, 50, 53, 56, 59, 63, and 67°C), respectively, for 3 min followed by cooling for another 3 min. The cells were then lysed by three cycles of freeze-thaw using liquid nitrogen. The soluble protein fractions were collected by centrifugation at 100,000 g for 20 min at 4°C and transferred into new ultracentrifuge micro-tubes. The protein concentrations of samples heated at 37 and 41°C were measured and used to normalize the load amount for immunoblot analysis. The solution protein was then mixed with loading dye and denatured at 95°C for 5 min. The remaining steps were consistent with the immunoblot analysis as described earlier.

For the ITDRF experiment, ALA was serially diluted from 32 μM to generate dose response curves. NCI-H460 cells were treated with each ALA concentration and one vehicle as a control in 50 μl of cell suspension in a 96-well PCR plate for 2 h at a cell incubator. The cell suspensions were then heated at 52°C for 3 min. The cell was lysed and the supernatant was isolated by ultra-centrifugation followed by immunoblot analysis with the procedures described earlier.

### Surface Plasmon Resonance Assay

The direct interaction between ALA and AKR1C1 was determined by an SPR assay using a Biacore T200 system (Cytiva, MA, United States) at 25°C based on a standard protocol. In brief, a nitrilotriacetic acid (NTA) sensor chip was first activated by injection of Ni^2+^ ions to capture the polyhistidine-tagged recombinant human AKR1C1 protein. When the signal baseline was stable, the different concentrations of ALA solution were successively injected to determine the binding affinity. The loading rate was 20 µl/min, and the times for association and dissociation were set as 240 and 300 s, respectively. The results were analyzed with TraceDrawer (Ridgeview Instruments ab, Sweden) in the 1:1 mode.

### AKR1C1, AKR1C2, and AKR1C3 Enzymatic Activity Assay

The effects of ALA on the activity of AKR1C1, AKR1C2, and AKR1C3 enzymes were investigated by detecting the rate of change in NADPH absorbance (*λ* = 340 nM). The assays were carried out in a 100-µl volume containing 2 µg recombinant proteins (Bio-Techne, Minnesota, United States), 1 mM s-tetralol, 0.5 mM NADP+ and 100 mM potassium phosphate buffer, pH = 7.4, at 37°C. To determine the inhibition activity of ALA, the 2 µg recombinant proteins in 50 µl of assay buffer were first incubated with DMSO or varying concentrations of ALA on ice for 10 min, and the reaction was started by adding 50 μL of substrate mixture (2 mM s-tetralol and 1 mM NADP+ in assay buffer). The absorbance of 340 nm was recorded immediately in the kinetic mode for 15 min. The relative enzymatic activity was normalized to that of the vehicle control group (DMSO) as 100%. All inhibition data were obtained from single experiments, with samples conducted in triplicate.

### Molecular Docking Study

The docking study of ALA with AKR1C1 was conducted by the AutoDock Vina program (version 1.1.2). First, the 3D-structure of ALA was generated by ChemBio3D and optimized under a minimization field using the MM2 forcefield, and then processed by Auto Dock Tools *via* adding hydrogens and assigning Gasteiger charges. AKR1C1 was extracted from the crystal structures (PDB ID: 1MRQ). For preparing receptors, the solvent molecules and original ligands from the crystals were removed, retaining one molecule of the respective enzyme and the NADPH cofactor. The binding pocket was defined to cover the original ligand within a 15 Å radius sphere. The docking experiments were carried out by taking into account only the flexibility of ligands. The docking conformation with the highest score was considered the binding conformation. Finally, PyMOL 2.5 (https://pymol.org) is used to visually analyze the docking results.

### Luciferase Reporter Assay

The inhibitory effect of ALA on STAT3 transcription activity was investigated by a luciferase reporter gene assay. NCI-H460 cells were transiently transfected with the pGM-STAT3 and pRL-TK-Luc plasmids. Transient transfections were performed with the Limitless^TM^ ELZ Fushion Kit (ELK biotechnology) according to the manufacturer’s instructions, and 24 h after transfection, the cells were preincubated with different concentrations of ALA for 1 h, and then treated with IL-6 (50 ng/ml) for another 6 h. Cells were collected and lysed, a luciferase assay was performed using the Dual Luciferase Reporter Gene Assay Kit (Beyotime, China) according to the manufacturer’s instructions, and luciferase activity was measured in a Spark 10M multimode microplate reader (TECAN, Sweden). Luciferase activity in different samples was normalized with protein concentration. The results are represented as the percentage of inhibition.

### Statistical Analysis

All biological data included in the manuscript are based on experiments with at least three biological replicates (*n*) with different numbers of technical replicates (N), given as mean values with either standard deviation (SD). The numbers of replicates and error types are indicated in the respective figure legends. After the test of homogeneity of variance, statistical analysis was performed using independent Student *t*-tests and analysis of variance (ANOVA) when variance was equal, whereas the Brown-Forsythe and Welch ANOVA tests were performed with unequal variance. Statistical significance was considered with a two-tailed *p* value under 0.05 (*α* = 0.05), and a highly significant difference was accepted when the *p*-value was less than 0.01.

## Results

### Identification of AKR1C1 as a Candidate Target of Alantolactone Through Proteome Integral Solubility Alteration Analysis

**FIGURE 1:**
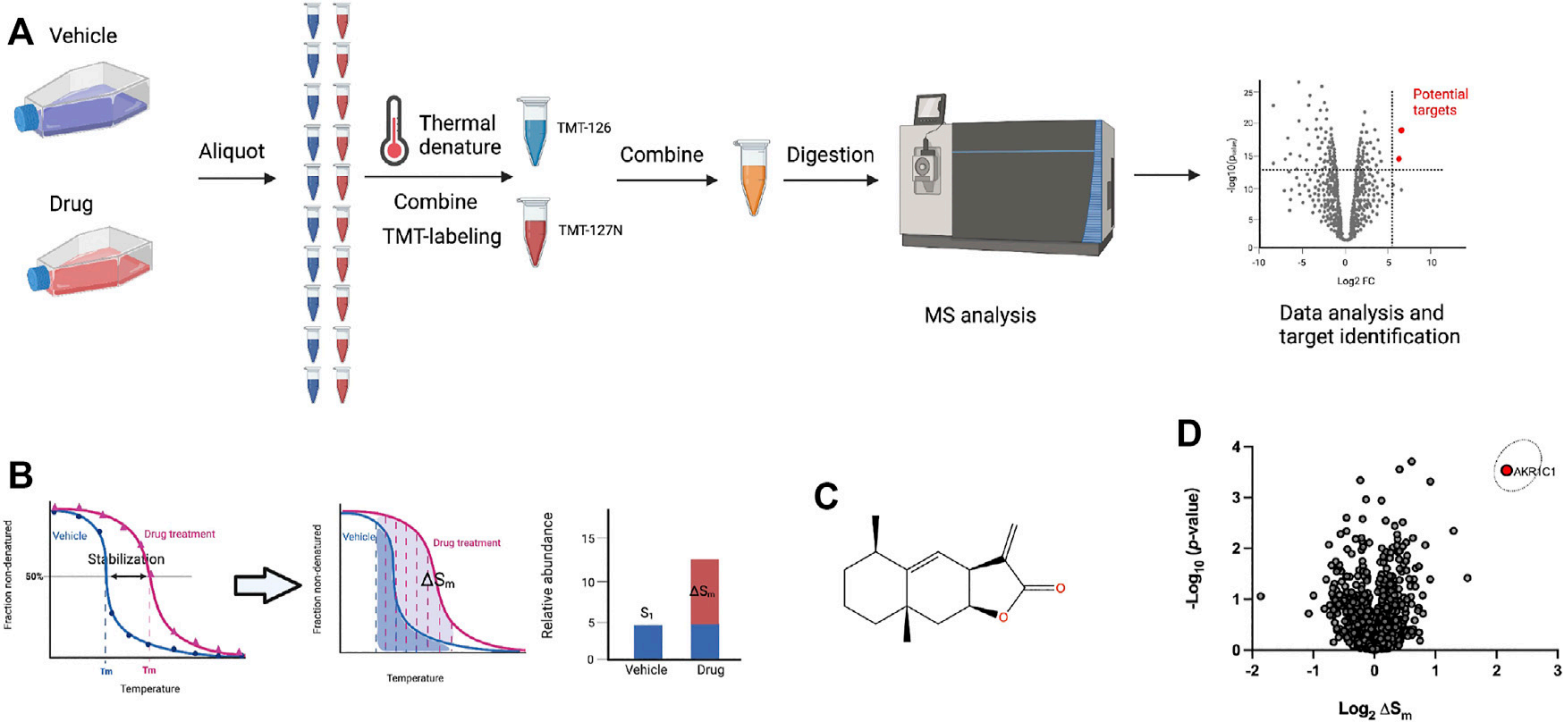
Target identification of ALA in NCI-H460 cells using PISA analysis. **(A)** Schematic workflow of the PISA experiment. Living NCI-H460 cells were treated with 10 μM ALA or DMSO for 2 h. Subsequently, aliquots were heated at 10 different temperatures for 3 min followed by a lysis by three cycles of freeze-thaw using liquid nitrogen. The 10 aliquots from the same group were then combined to one sample, and the soluble protein fractions were collected by an ultra-centrifugation. After TMT labeling, the samples were analyzed by mass spectrometry. **(B)** PISA concept of pooling together individual samples corresponding to different temperature points and thus hardware integration of the melting curve without detailed determination of its shape, and measuring ΔS_m_ along with statistical *p*-value could be used as a difference between the integral abundances of the protein in the treated and untreated samples. **(C)** The chemical structure of ALA. **(D)** PISA analysis results after ALA treatment was plotted by the volcano plot showing the −log_10_ (*p*-values) *versus* the log_2_ (ΔS_m_). The ΔS_m_ (fold change) and *p*-value were calculated from three biological replicates of ALA treatment compared with solvent control group. Data were preprocessed using MaxQuant and plotted by Prism. The original data for Prism was provided in Supplementary Table S2.

Since the direct targets of ALA for its anticancer potential are still unclear and molecular target identification is an essential step for understanding the underlying mechanism of action for a drug’s activity, we first performed the PISA assay for the unbiased identification of direct molecular targets of ALA (Figure 1A). This method is modified from TPP, which employs the integral of the area under its melting curve instead of the determination of melting temperature by plotting the melting curve (Figure 1B; Gaetani et al., 2019). Thus, an increase in the abundance of a protein with the treatment of ALA suggests the target-ALA binding. NCI-H460 cells were incubated with ALA (10 μM) or equal volumes of DMSO for 2 h and subsequently heated at a range of temperatures from 37 to 65°C to induce thermal denaturation. The cells were lysed through three freeze-thaw cycles in liquid nitrogen, and the soluble proteins were then obtained by ultracentrifugation. All samples from the same group were denatured at different temperatures, pooled together, tryptic-digested, and labeled using isobaric tandem mass tag (TMT) labels. The labeled peptides from different groups were then mixed, and finally subjected to analysis by Orbitrap mass spectrometry.

The quantitative data were analyzed by MaxQuant to obtain the total abundance of the soluble protein after treatment with ALA or solvent control. The fold changes (ΔS_m_) were calculated from three biological replicates of the ALA treatment compared with solvent control groups, and the volcano plot was used to visualize the statistical significance *versus* magnitude of change. In order to ensure the accuracy of this experiment, methotrexate, known to target dihydrofolate reductase (DHFR), was used as a positive control drug that was analyzed in parallel with ALA (Morales et al., 2009). The results of methotrexate were first analyzed to check whether DHFR would stand out in the PISA assay. As expected, DHFR was significantly stabilized and clearly shown in the results of the PISA assay (Supplementary Figure S1), which suggested credible results from this experiment. With regard to the results of ALA, as shown in Figure 1C, the volcano plot showed the −log_10_ (*p*-values) *versus* the log_2_ (ΔS_m_), and it was quite apparent that AKR1C1 was a strong positive outlier at the extreme top right corner of the ΔS_m_ distribution (ΔS_m_ = 4.48, *p* = 0.0003), indicating AKR1C1 as a top target candidate of ALA in NCI-H460 cells. The detailed quantitative mass spectrometry and PISA analysis data are provided in Supplementary Table S1.

### Direct Interaction Between ALA and AKR1C1 Protein

**FIGURE 2:**
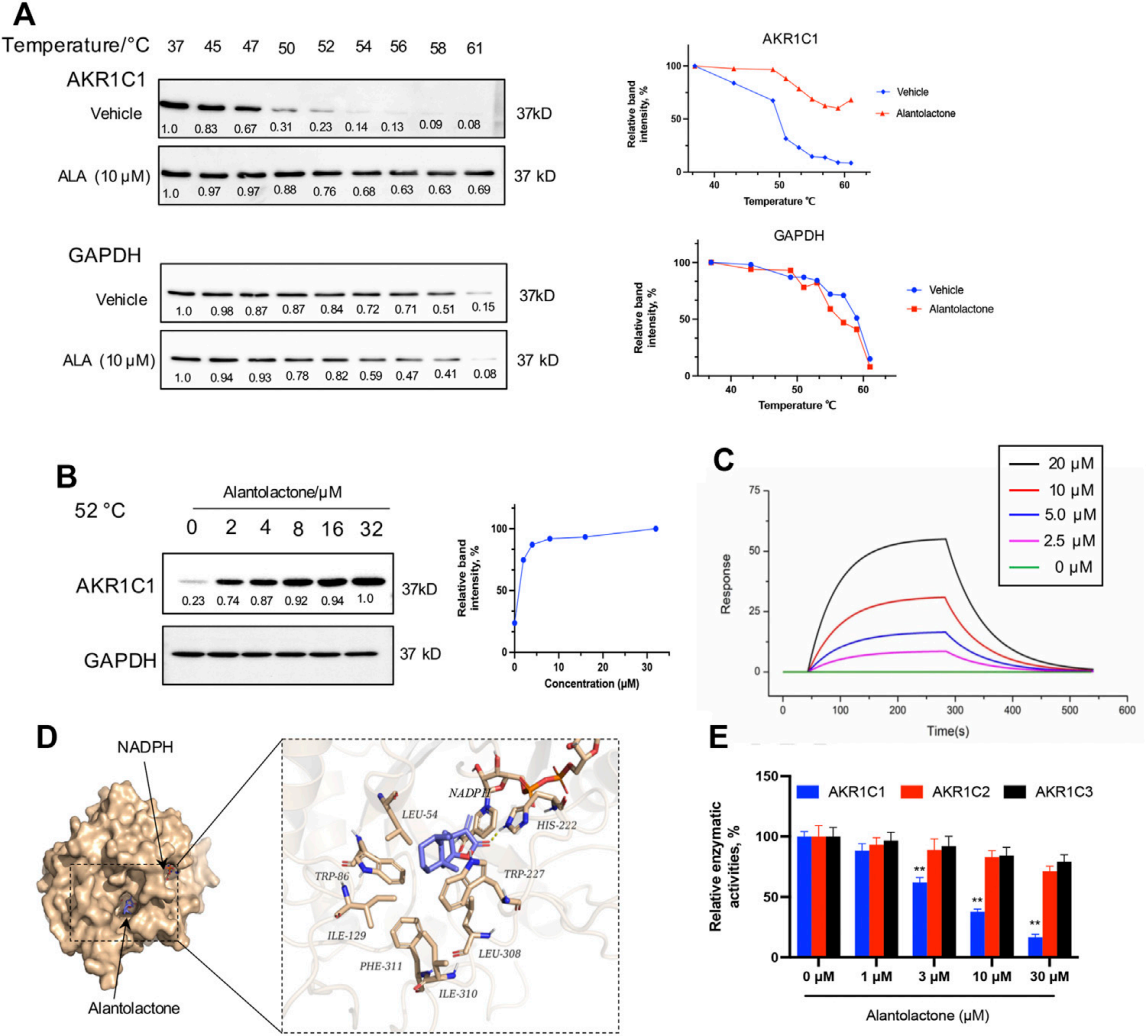
Target verification of direct binding between ALA and AKR1C1. **(A)** Thermal stabilization of AKR1C1 by ALA (10 μM) in intact NCI-H460 cells was assessed via CETSA analysis. GAPDH was used as control. The melting curves for AKR1C1 and tubulin in NCI-H460 cells in the presence of DMSO (blue curve) and 10 μM ALA (red curve) were plotted by the corresponding mean gray values. All data were normalized to the response observed at each condition at 37°C. **(B)** Dose-dependent stabilization of AKR1C1 by ALA in intact ALA cells was examined at 52°C by ITDRF analysis. GAPDH was used as control. The dose-response curve was generated by the quantification based on mean gray values. All data were normalized to the intensity at the treatment of 32 μM ALA. **(C)** SPR bio-sensor was used to detect the binding of ALA to AKR1C1. Apparent K_D_ value is calculated by SPR data. The fitted K_D_ is 11.8 μM. **(D)** The binding mode of ALA to AKR1C1·NADPH as determined by molecular docking. Left, structural overview of docked complex. The protein was shown in a surface representation in light yellow, while ALA and NADPH were shown in a stick representation. Right, zoom-in view of the predicted AKR1C1–ALA interface. The forming hydrogen bond with His-222 was shown as yellow dash. And residues around the predicted binding pocket, including Leu-54, Trp-86, Ile-129, Phe-311, Ile-310, Leu-308, and Trp227, were labeled. **(E)** The relative catalytic activity of AKR1C1, AKR1C2, and AKR1C3 upon treatment with different concentrations (as indicated) of ALA. Data were presented as mean ± SD (*n* = 3), **p < 0.01, vs control. Uncropped blots are provided in Supplementary Figure S3.

In this section, the results obtained from the PISA assay were validated, and we investigated whether ALA directly interacts with the AKR1C1 protein. First, the thermal stabilization of AKR1C1 by ALA was determined by CETSA. NCI-H460 cells were exposed to 10 μM ALA for 2 h and subjected to CETSA according to the protocol described in the methodology. The immunoblotting analysis showed a highly reproducible thermal stabilization, similar to that observed through the PISA assay. Treatment of ALA led to a significant shift of melting point of AKR1C1 from 50.3°C to over 62°C (Figure 2A), while no change was observed for GAPDH, indicative of cellular engagement of ALA to AKR1C1 in NCI-H460 cells. Moreover, an isothermal dose-response fingerprinting (ITDRF) experiment was performed at 52°C with varying concentrations of ALA treatment. The exposure to ALA in NCI-H460 cells showed a dose-dependent stabilization of AKR1C1 (Figure 2B).

To further assess the direct interaction of AKR1C1 with ALA, the binding property of recombinant AKR1C1 to ALA was investigated using surface plasmon resonance (SPR). SPR is an optical technique without any labeling for measuring the protein–ligand interaction based on the change in the refractive index (Englebienne et al., 2003). As a result, we found that ALA was bound to AKR1C1 with a binding affinity in the micromole range. The value of the equilibrium dissociation constant (K_D_) was 11.8 μM (Figure 2C). In order to gain more precise binding information, we used molecular docking to predict the potential ALA binding sites in AKR1C1. As shown in Figure 2D, the virtual docking profile demonstrated that ALA binds at the active pocket of AKR1C1, where the compound is close to cofactor NADPH (reduced nicotinamide adenine dinucleotide phosphate). The ketone group of ALA is predicted to form a hydrogen bond with the catalytic residue His-222. The other residues, including Leu-54, Trp-86, Ile-129, Phe-311, Ile-310, Leu-308, and Trp227, form hydrophobic interactions with the alkyl moiety of ALA. The calculated binding free energy (delta G) of the ALA-AKR1C1 complex is −8.12 kcal/mol.

### Alantolactone Selectively Inhibits AKR1C1

AKR1C1 is known as the aldo-keto reductase which is a crucial enzyme for the conversion of aldehydes and ketones to their corresponding alcohols with NADPH as a cofactor (Zeng et al., 2019). To investigate the selectivity of ALA for AKR1C1, we next performed the enzymatic assay of human aldo-keto reductase family C *in vitro*, monitoring AKR1C (AKR1C1, AKR1C2, and AKR1C3)-catalyzed conversion of s-tetralol as substrate, to record the generation of NADPH as a readout. Figure 2E showed that ALA potently and selectively inhibited the catalyzed activity of AKR1C1 in a dose-dependent manner. Each experiment was repeated three times, and the differences among the means were significant as determined by the Student t-test (∗∗*p* < 0.01).

### High AKR1C1 Expression is Associated With Metastasis and Poor Prognosis in Non-Small-Cell Lung Cancer Patients

**FIGURE 3:**
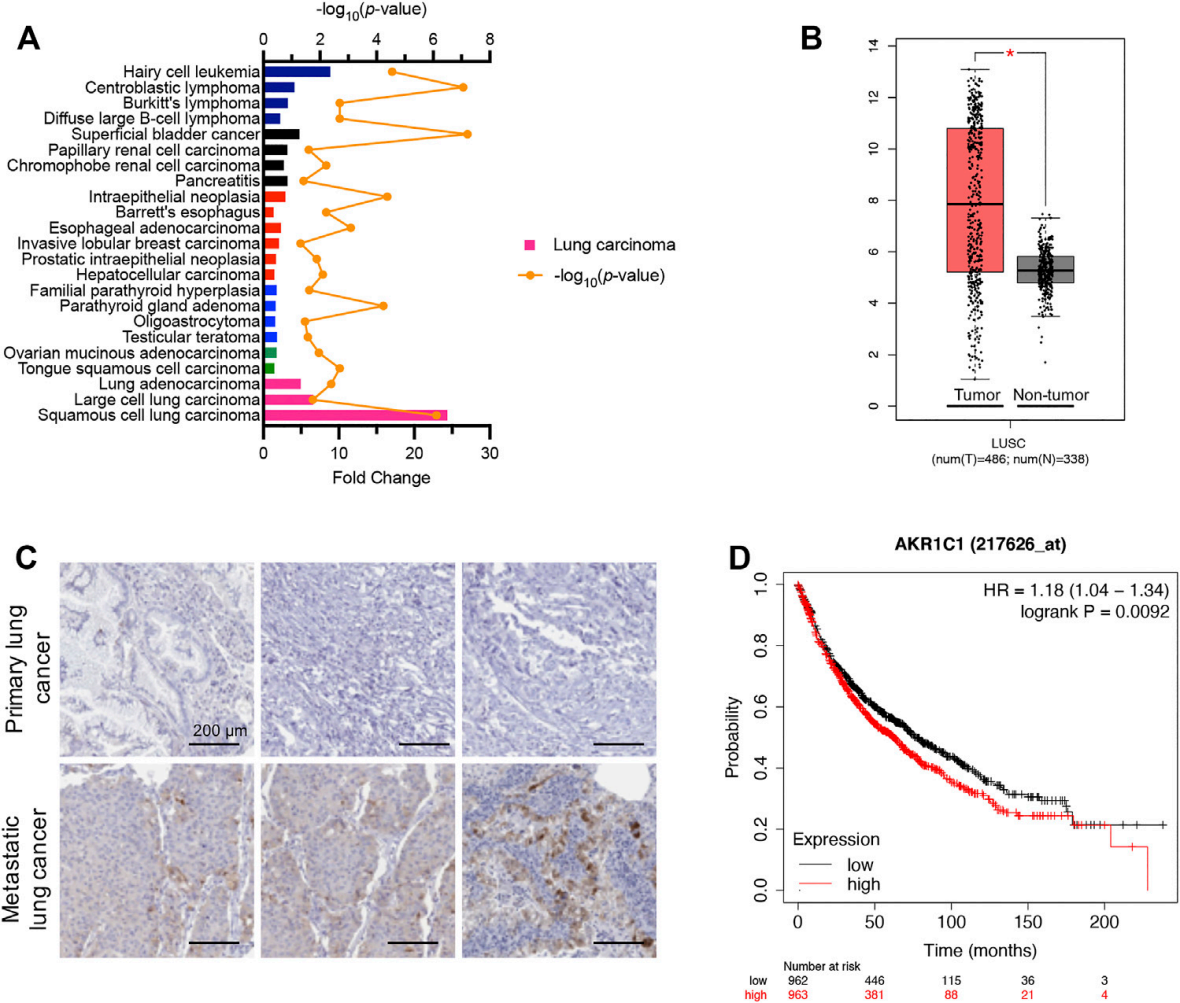
High expression levels of AKR1C1 were observed in NSCLC clinical samples, particularly in metastatic tumors. **(A)** High mRNA fold change of AKR1C1 was observed among different cancer types in clinical samples, particularly in lung carcinoma. Data were obtained from the Oncomine database. https://www.oncomine.org. **(B)** AKR1C1 is upregulated in lung cancer samples compared with normal lung samples. The box plots of 486 lung cancer samples (red) and 338 normal samples (gray) revealed upregulation of AKR1C1 in lung cancer compared with normal tissue. Data were analyzed using the GEPIA database (http://gepia.cancer-pku.cn/); *p < 0.05. **(C)** Immunohistochemical staining showed that the expression levels of AKR1C1 were significantly upregulated in metastatic tumors compared with corresponding primary tumors obtained from the same NSCLC patient. **(D)** The effects of AKR1C1 on the overall survival of NSCLC patient. The data were analyzed by the Kaplan–Meier analysis. Lung Cancer. http://kmplot.com/analysis/index.php?p=service&cancer=lung.

According to the Oncomine database (https://www.oncomine.org) (Rhodes et al., 2004), which displays the expression profiles of multiple types of cancer compared to corresponding normal tissues, the AKR1C1 mRNA levels in cancer samples were significantly higher than those in normal tissues, especially in lung carcinoma samples (Figure 3A). As shown, the squamous cell lung carcinoma, large-cell lung carcinoma, and lung adenocarcinoma were determined to be 24.36-fold, 6.59-fold, and 4.95-fold, respectively. This finding was supported by GEPIA (Gene Expression Profiling Interactive Analysis) (Li et al., 2021), which showed that AKR1C1 was significantly higher in NSCLC (Figure 3B). Thus, we were encouraged to speculate that AKR1C1 might play a critical role in the cancer initiation and progression of NSCLC.

To identify the relationship between AKR1C1 and NSCLC metastasis, we examined AKR1C1 expression in three pairs of clinical NSCLC and corresponding adjacent non-tumor tissue samples. The immunohistochemical assay showed that the expression level of the AKR1C1 protein in metastatic tumors was significantly elevated compared to the corresponding primary tumors (Figure 3C), suggesting that higher AKR1C1 expression is associated with metastasis in NSCLC. Moreover, the relationship between AKR1C1 and the survival time of lung cancer patients after surgery was investigated. We found that the overall survival time of patients with high levels of AKR1C1 was cut down compared with patients with low levels of AKR1C1 (*p* = 0.0092) by the Kaplan–Meier analysis, suggesting the specific role of AKR1C1 in cancer metastasis (Figure 3D).

### Alantolactone Inhibited Cell Proliferation and Metastasis in NCI-H460 Cells

**FIGURE 4:**
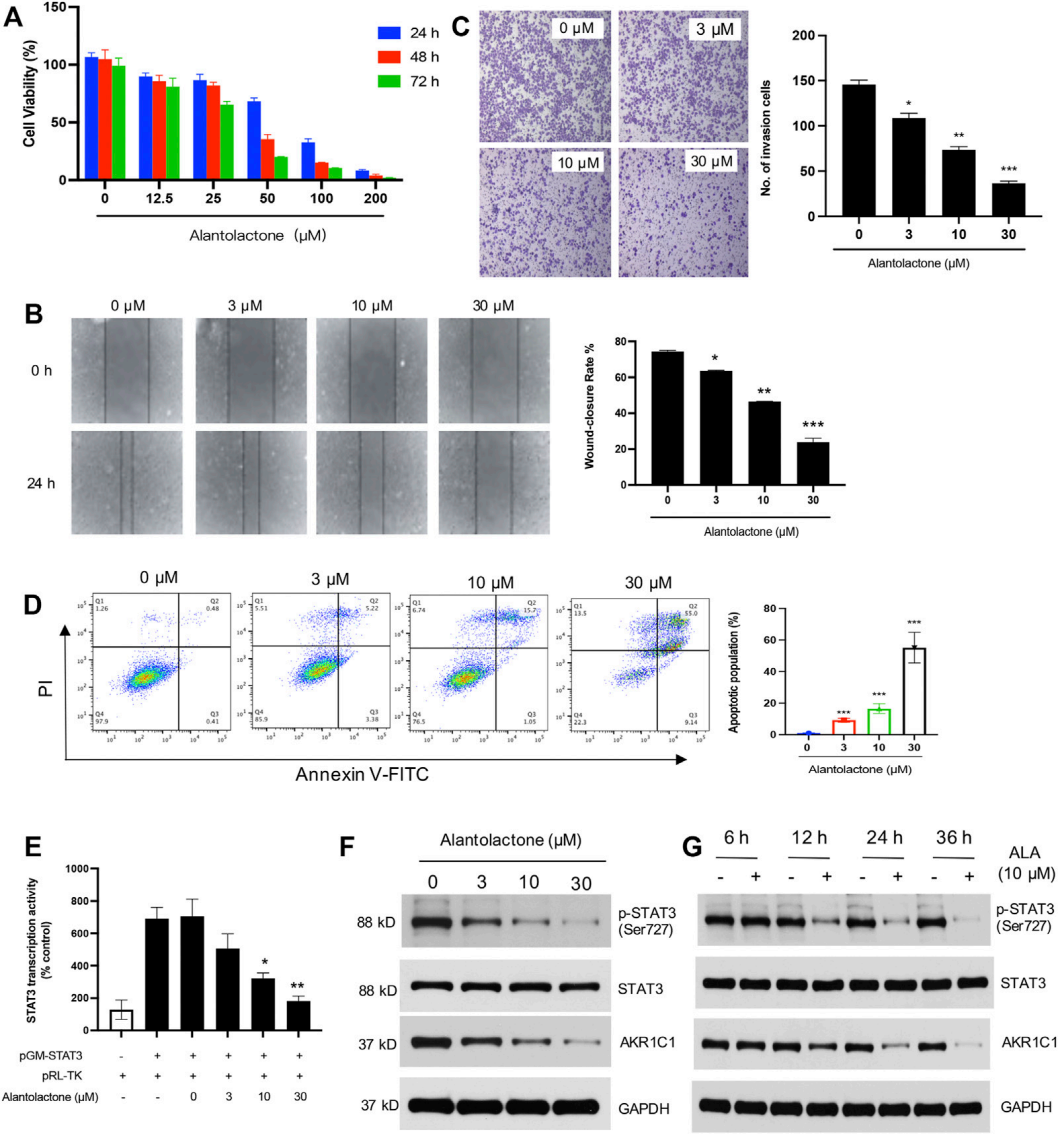
Anticancer activity of ALA *in vitro*. **(A)** ALA inhibits the proliferation of NCI-H460 cells in a dose-dependent manner. Cell viabilities were determined using the CCK-8 assay. **(B)** The effects of ALA on migration potential of NCI-H460 cells were examined using cell wound healing assay. NCI-H460 cells were seeded and treated with ALA at indicated concentrations (3, 10, and 30 μM) for 24 h. The cell-free gaps were respectively photographed at 0 and 24 h on each plate. Quantitative analysis of the gap distance in the wound healing assay. *p < 0.05, **p < 0.01, ***p < 0.001, vs control. **(C)** The effects of ALA on invasion potential of NCI-H460 cells were examined using Transwell assay. NCI-H460 cells were pretreated with ALA at indicated concentrations for 24 h and seeded in the Transwell insert (upper chambers were pre-coated with ECM gel). The cells that passed the Transwell insert membrane were stained with crystal violet and counted under a light microscope. Quantitative analysis of the migrated number of cells in the Transwell invasion assay. *p < 0.05, **p < 0.01, ***p < 0.001, vs control. **(D)** The apoptosis cells were assessed using flow cytometry assays. The number of cells undergoing apoptosis in the ALA treatment group was significantly higher than number of cells in the control group. ***p < 0.001 vs. control. **(E)** ALA inhibits STAT3 transcriptional activity in human NCI-H460 cell lines. NCI-H460 cells were transfected with pGM-STAT3 and pRL-TK-Luc plasmids, and the impact of different concentrations of ALA on the luciferase activity was determined. Data were presented as mean ± SD, *p < 0.05, **p < 0.01, vs untreated control. **(F,G)** Western blot analysis displayed that ALA inhibited the expression of AKR1C1 and phosphorylation of STAT3 in the time- and dose-dependent manners. GAPDH was used as loading control.

AKR1C1 acts as an important inducement in the proliferation and migration of lung cancer cells (Tian et al., 2016); the cytotoxicity of ALA was thus examined in human NSCLC cells. The NCI-H460 cells were treated with varying concentrations of ALA for 24, 48, and 72 h. As shown in Figure 4A, the cell viability was determined by the CCK-8 assay and the results demonstrated that ALA inhibited the proliferation of NCI-H460 cells in the dose- and time-dependent manners. It was noticeable that a 24-h treatment of ALA did not show a significant reduction (<20%) of cell viability when the concentration was lower than 25 µM. Next we investigate whether ALA inhibited the cell migration and invasion in subsequent experiments; hence, concentrations of ALA no more than 30 µM were used to prevent interference from the cytotoxic effects.

Cancer cell migration into the surrounding tissue is an important step involved in the metastasis process (Pijuan et al., 2019). We next assessed the effects of ALA on the cell migratory behavior of NCI-H460 cells. By performing a wound healing assay, we found that the wound healing rate showed a significant decrease with the treatment of ALA in a dose-dependent manner when the concentration was over 3 µM, suggesting an inhibitory effect of ALA on NCI-H460 cell migration (Figure 4B). Moreover, a Transwell invasion assay was conducted to evaluate the effect of ALA on cell invasion. As displayed in Figure 4C, the number of invaded cells crossing the Matrigel-coated membrane was shown to have observable reduction after the treatment of 10 and 30 µM ALA, with an inhibition rate of 49.5 and 74.9%, respectively. Collectively, these results indicated that ALA inhibited cell proliferation and the metastatic potential of NCI-H460 cells.

### Alantolactone Induced Cell Apoptosis and Inhibited Phosphorylation of STAT3 in NCI-H460 Cells

The rates of cellular apoptosis were assessed in NCI-H460 cells by flow cytometry. The results demonstrated the numbers of early and late apoptotic cells at 48 h post-treatment with different concentrations of ALA in NCI-H460 cells (Figure 4D). Compared with the untreated control group, the numbers of early and late apoptotic cells in the ALA treatment group were significantly increased (*p* < 0.001). The apoptotic populations of NCI-H460 cells after the treatment of 3 µM, 10µM, and 30 µM ALA were determined as 8.6, 16.7, and 64.1%.

AKR1C1 is also a key component for the phosphorylation of STAT3 and it could facilitate the interaction of STAT3 with its upstream kinase JAK2, which promotes tumor metastasis in NSCLC (Hong et al., 2018). As one of the most known oncogenic transcription factors, STAT3 plays an important role in the transcription of its downstream target genes (Yang et al., 2005; Yu et al., 2014). We first assessed the effects of ALA on the activated form of STAT3 (phospho-STAT3), and the inhibitory effect of ALA on STAT3 transcription activity was investigated by a luciferase reporter gene assay. As shown in Figure 4E, we found that the treatment of ALA significantly decreased the transcriptional activity of STAT3. The Western blot results further displayed that the protein levels of phosphorylated STAT3 were significantly decreased in the ALA treatment group compared with the control group (Figures 4F, G), and the inhibition effect was shown in the dose- and time-dependent manners. Importantly, the expression level of AKR1C1 was also obviously reduced after the treatment of ALA. It is consistent with previous findings that ALA directly targets AKR1C1 and acts as an AKR1C1 inhibitor.

### Alantolactone Inhibited Tumor Growth in a Xenograft Mouse Model *In Vivo*

**FIGURE 5:**
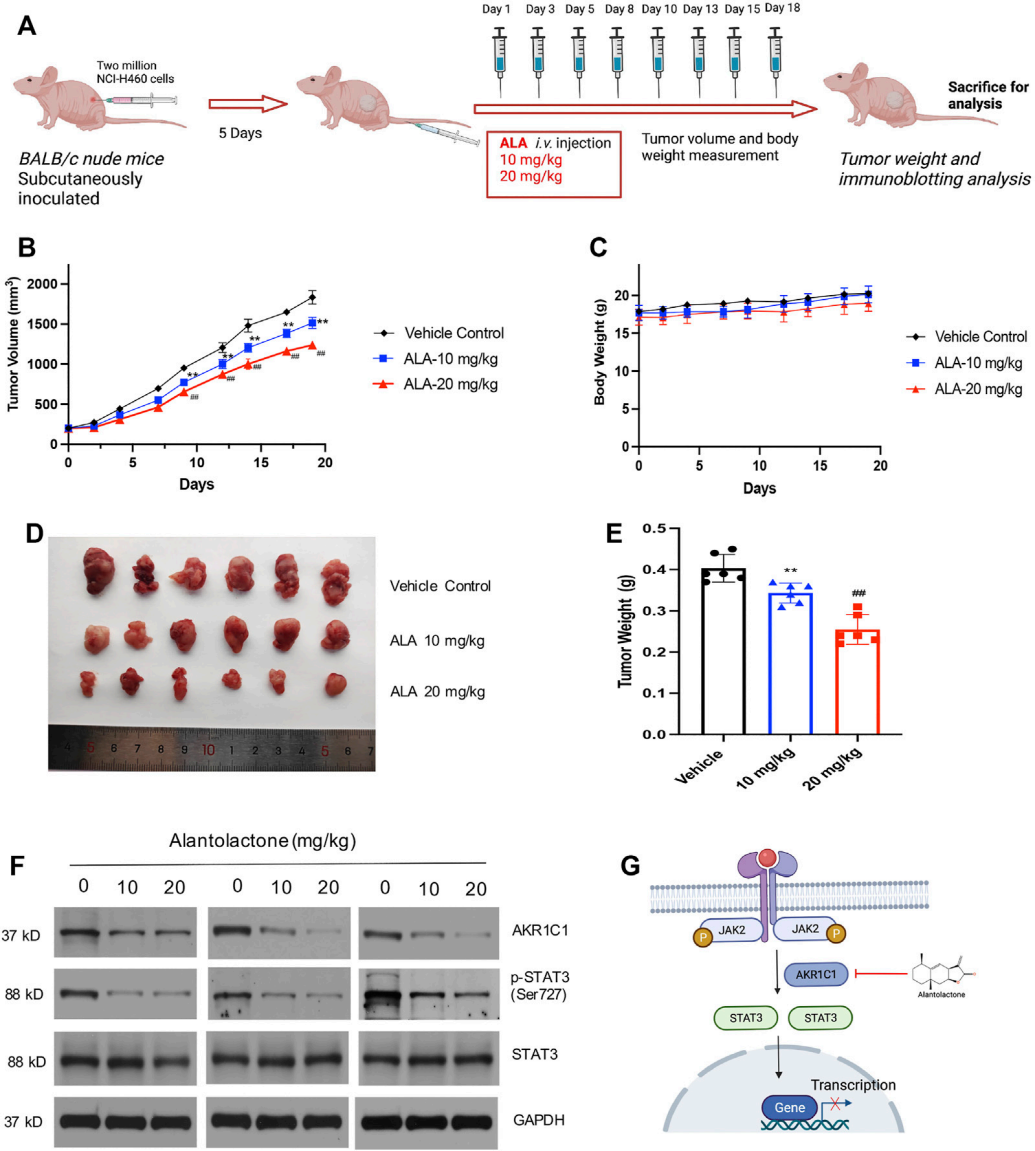
ALA treatment suppresses tumor growth *in vivo*. **(A)** Scheme of *in vivo* ALA treatment in the NCI-H460 cell xenograft model. Two million NCI-H460 cells were subcutaneously inoculated, and after 5 days, mice were injected with DMSO or ALA (10 and 20 mg/kg in DMSO) intravenously (*i.v.*) at days 1, 3, 5, 8, 10, 13, 15, and 18. At day 19 of treatment, mice were sacrificed for tumor weight and immunoblotting analysis. **(B)** ALA inhibited tumor growth in NCI-H460 xenograft models. Average and SD of the tumor volumes (mm^3^) *versus* time. NCI-H460 cells were injected subcutaneously into mice (*N* = 6/group), and then mice were treated with or without ALA (10 and 20 mg/kg) every 2 days for 21 days. Tumor sizes were measured every 2 days. **p < 0.01, ##*p* < 0.01. vs vehicle. **(C)** Averages and SDs of nude mouse weights versus the time (mean weight ± SD; *n* = 6/group). **(D)** Images of xenograft tumor tissues dissected from each mouse at the end of experiments. **(E)** Mean weight of tumors were measured. Data were presented as mean ± SD, **p < 0.01, ##*p* < 0.01. vs vehicle. **(F)** Expression of AKR1C1 and phosphorylation of STAT3 in the tumor tissues were detected by immunoblot. GAPDH was used as loading control. **(G)** Schematic overview of the mechanism of ALA in NSCLC cells. ALA directly binds to AKR1C1, decreases the downstream phosphorylation of STAT3, and ultimately, this process leads to the death of the NSCLC cells.

Finally, the subcutaneous NCI-H460 cell xenograft tumors were established in BALB/c nude mice to determine the antitumor activity of ALA *in vivo*. The Balb/c nude mice (8 weeks old) were subcutaneously inoculated with two million NCI-H460 cells. When the mean tumor size reached approximately 200 mm^3^, ALA was administered by tail intravenous injection every two or three days at 10 mg/kg (*n* = 6) and 20 mg/kg (*n* = 6) for 18 days, while the control group received an equal volume of vehicle (Figure 5A). As a result, starting from Day 9, tumor volumes from both treatment groups were significantly smaller than the vehicle group (*p* < 0.01) (Figure 5B). In detail, the mean tumor volume of the 10 mg/kg ALA-treated group at end point was 1514.08 ± 68.24 mm^3^, whose tumor growth inhibition (TGI) was approximately 17.4% compared with that of the vehicle control group (1,833.92 ± 85.14 mm^3^, *p* < 0.01). As for the 20 mg/kg ALA-treated group, its mean tumor volume was 1,238.00 ± 52.86 mm^3^ with a more significant TGI of 32.5% (*p* < 0.01). Moreover, it is noteworthy that these two effective dosages of ALA were well tolerated as body weights were stable in both dose groups across the studies (Figure 5C). At the end of the experiment, the representative images of tumor tissues from all groups are displayed in Figure 5D. The tumor weights were measured and showed a similar reduction to those of tumor volumes upon the treatment of ALA (Figure 5E). Furthermore, we examined the effect of the inhibitor on expression levels of AKR1C1 and STAT3 in xenograft tumors. As expected, AKR1C1 and phosphorylated STAT3 proteins are observably decreased in the ALA-treated groups (Figure 5F, *n* = 3). The level of STAT3 remains relatively steady in different samples. These results are consistent with the observations made in the NCI-H460 cell *in vitro* experiments.

## Discussion

As a valuable source for novel drug discoveries, screening the natural products extracted from medical plants is always considered the best shortcut (Atanasov et al., 2021). ALA, a sesquiterpene lactone compound, has been extracted from the plants for decades and has been shown to have a variety of pharmacological activities (Gierlikowska et al., 2020; Shi et al., 2020; Chen et al., 2021; Sun et al., 2021), but little is known about its direct target(s). Inspired by its remarkable anticancer activity, we have performed a PISA analysis to identify the protein targets of ALA in cellular conditions. In this study, we found that AKR1C1, which is a member of the aldo-keto reductase family, is directly bound and selectively inhibited by ALA. The inhibition of AKR1C1 by ALA results in the deactivation of STATs and the suppression of cell proliferation and metastasis in NSCLC.

Although there are various methods that are available for target identification, PISA derived from TPP is the most suitable approach for natural products as it does not require any chemical modification to design a probe (Gaetani et al., 2019). Through monitoring of changes in protein thermal stability across the whole proteome using multiplexed quantitative mass spectrometry, the target engagement of drugs in living cells would be identified in an unbiased manner (Peuget et al., 2020; Corrales et al., 2021; Wilke et al., 2021). Notably, PISA employs the integral of the area under its melting curve instead of the determination of melting temperature by plotting the melting curve, which could significantly improve the low-throughput drawback of melting curve fitting in TPP (Li et al., 2020). Therefore, PISA analysis could be a valuable tool to discover the protein targets of a small molecule with specific bioactivity, which will give mechanistic insights to explain the bioactivity of ALA in anticancer and provide opportunities of developing novel sesquiterpene lactone-based AKR1C1 inhibitors for pharmacological treatment of NSCLC.

Several strategies have been exploited to suppress cell proliferation and metastasis to treat and prolong the survival time of patients with NSCLC, including targeting the metastasis microenvironment (Binnewies et al., 2018), using drugs that target tumor-driving oncoproteins (Mok et al., 2017; Wu et al., 2020), targeting immune checkpoint receptors and ligands (Hellmann et al., 2017; Ribas and Wolchok, 2018), and targeting mediators of metastatic plasticity (Pascual et al., 2017). AKR1C1 regulates the metabolism of progesterone and is closely related to the progression of malignant tumors (Tian et al., 2016; Wei et al., 2021). Overexpression of AKR1C1 is shown to play an important role in tumor invasion, metastasis, and drug resistance (Hsu et al., 2001; Tian et al., 2015). According to our analysis data of mRNA levels from the Oncomine Database and GEPIA, AKR1C1 in lung cancer samples had a significantly higher expression (Figures 3A–C). Importantly, we found that the overall survival time of patients was inversely correlated with the expression levels of AKR1C1 by the Kaplan–Meier analysis (Figure 3D). Consequently, AKR1C1 could be used as a new target for the clinical treatment of NSCLC.

In this regard, our discovery of ALA as a direct natural inhibitor of AKR1C1 with selectivity in NCI-H460 cells provides a unique pharmacological candidate to inhibit cell proliferation and metastasis in NSCLC. In this docked complex, the α,β-unsaturated ketone of the ALA was in close proximity to the cofactor and the unsaturated lactone ring of the ALA molecule was directed toward the 4-pro-R hydrogen of NADPH (Figure 2D). This docked conformation is regarded as “productive” since hydride transfer from the NADPH is possible for the steroid in this location (Jin and Penning, 2006). Hydrophobic interactions with residues Leu-54, Trp-86, Ile-129, Phe-311, Ile-310, Leu-308, and Trp227, and hydrophilic interactions (hydrogen bond) with His222 further stabilized the ligand. Compared with the overexpression of AKR1C1 in NCI-H460 cells, ALA treatment resulted in a selective inhibition of AKR1C1 activity and the expression level of AKR1C1. ALA inhibited cancer cell growth both in the cell model and the murine xenograft model. We thus believe that ALA may serve as an ideal candidate drug for the development of anticancer therapies for NSCLC.

Based on previous studies, ALA is always used as the STAT3 inhibitor that blocks the phosphorylation of STAT3 (Khan et al., 2013; Chun et al., 2015). Our work also demonstrated that STAT3 activation was significantly inhibited upon ALA treatment, both in the cell and tumor samples, as expected (Figures 4F,G, 5F). An increasing number of studies have provided strong evidence that AKR1C1 is a key component in the STAT3 pathway, which is critical for metastasis in different cancer models (Hong et al., 2018; Zhu et al., 2020; Wang et al., 2021). AKR1C1 interacts with STAT3 and promotes its phosphorylation, so as to promote the expression of downstream-related genes and finally promote tumor metastasis (Chang et al., 2019; Yang et al., 2019). From the results of PISA, several proteins, including RPS2, RPL35, RPL31, and RPL7, showed decreased stabilities upon treatment of ALA (Figure 1D; Supplementary Table S2). All of them belong to ribosomal proteins, which are involved in the cellular process of translation (Mao-De and Jing, 2007). It is consistent with the inhibition of STAT3 to block transcription of target genes. Consequently, we proposed that the inhibition of STAT3 induced by ALA treatment should be caused by the direct binding of AKR1C1, the upstream regulator of STAT3. ALA binds to AKR1C1 and shows strong suppression effect against AKR1C1, resulting in an inhibition of STAT3, to inhibit cancer proliferation and metastasis.

In summary, based on the previous findings that AKR1C1 plays a crucial role in cell proliferation and metastasis in NSCLC, and overexpression of AKR1C1 is related to poor prognosis in NSCLC patients, our studies convincingly demonstrate that using small-molecule ALA to target AKR1C1, leading to the inactivation of its downstream STAT3 signaling pathways, is feasible with regard to the suppression of the proliferation and metastasis of NSCLC (Figure 5G). To this end, our study implies that AKR1C1 is a potential therapeutic target for NSCLC and using ALA to target AKR1C1 would be a promising strategy to combat NSCLC in future therapies.

## Data Availability

The datasets presented in this study can be found in online repositories. The names of the repository/repositories and accession number(s) can be found below: ProteomeXchange Consortium via the PRIDE partner repository with the dataset identifier PXD031082. Further information and requests for resources and reagents should be directed to and will be fulfilled by the lead corresponding author CS, 29136909@qq.com.
