# Supplementary material for: Natural Product Alantolactone Targeting AKR1C1 Suppresses Cell Proliferation and Metastasis in Non-Small-Cell Lung Cancer
Source: Front Pharmacol. 2022 Mar 15;13:847906. doi: 10.3389/fphar.2022.847906 (PMC8965451; doi:10.3389/fphar.2022.847906)

# Supplemental information

## A small-molecule inhibitor targeting AKR1C1 suppresses cell proliferation and metastasis in non-small cell lung cancer

Zhiwen Fu[1,2], Shijun Li[1,2], Jinmei Liu[1,2], Cong Zhang[1,2], Chen Jian[1,2], Lulu Wang[1,2], Yu Zhang[1,2,] *, Chen Shi[1,2, 3,] *

1 Department of Pharmacy, Union Hospital, Tongji Medical College, Huazhong University of Science and Technology, Wuhan, 430022, China

2 Hubei Province Clinical Research Center for Precision Medicine for Critical Illness, Wuhan, 430022,China

3 Lead Contact

* Correspondence: whxhzy@163.com (Y. Zhang); 29136909@qq.com (C. Shi).

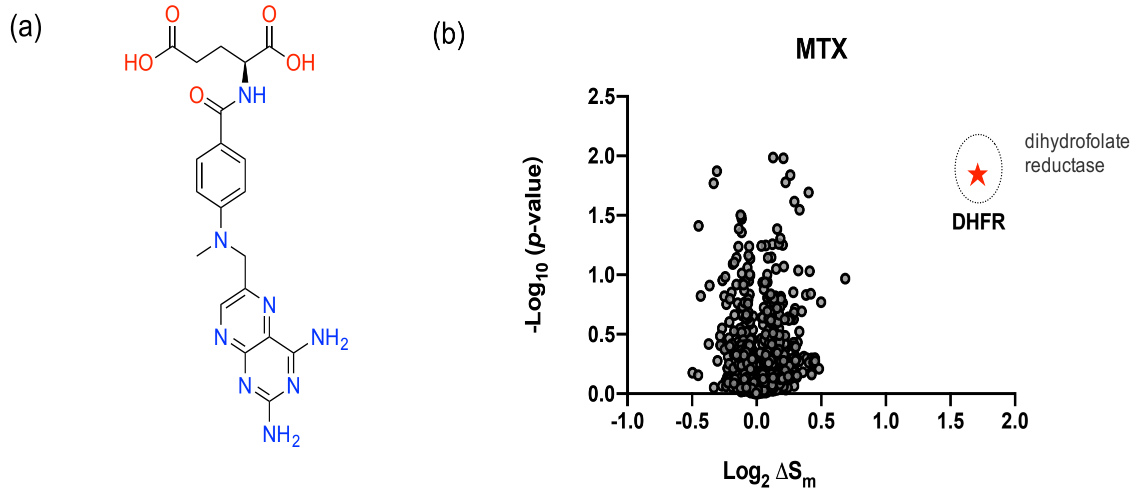


**Supplementary Figure S1**. Dihydrofolate reductase (DHFR) was identified as a molecular target of methotrexate (MTX). (a) The chemical structure of MTX; (b) The thermal stability of DHFR was shown to be the most significant increase one in the volcano plot.

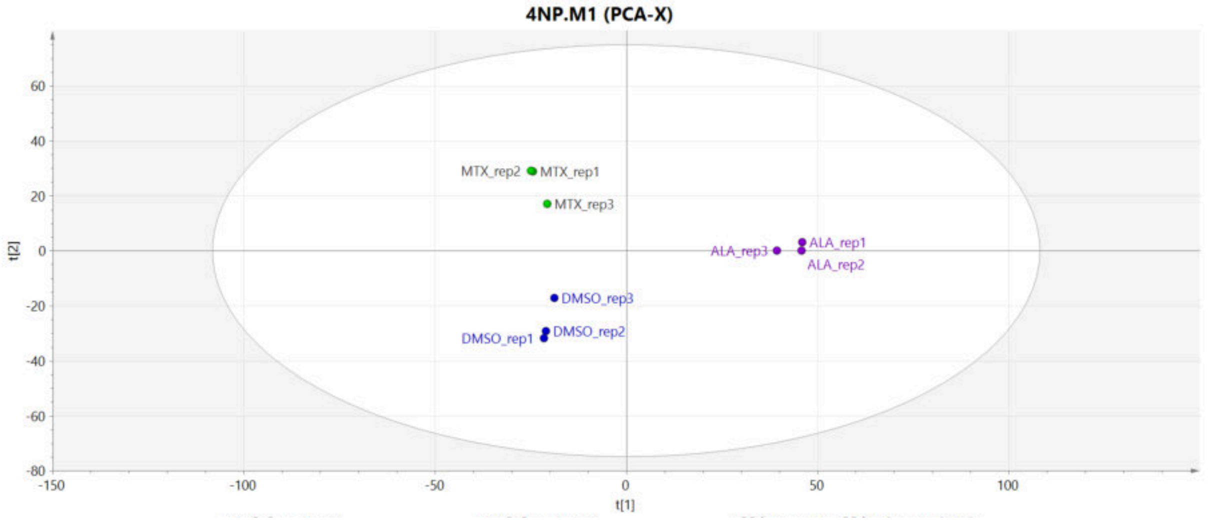


**Supplementary Figure S2**. Multivariate analysis of the protein abundance from LC-MS/MS. The Principal component analysis (PCA) was performed using the protein abundance values from LC-MS/MS to obtain PCA loading plot that explained the total data variation.

# Original western blot data:

**S1. CETSA experiment (two blots from two groups exposed at same time )**

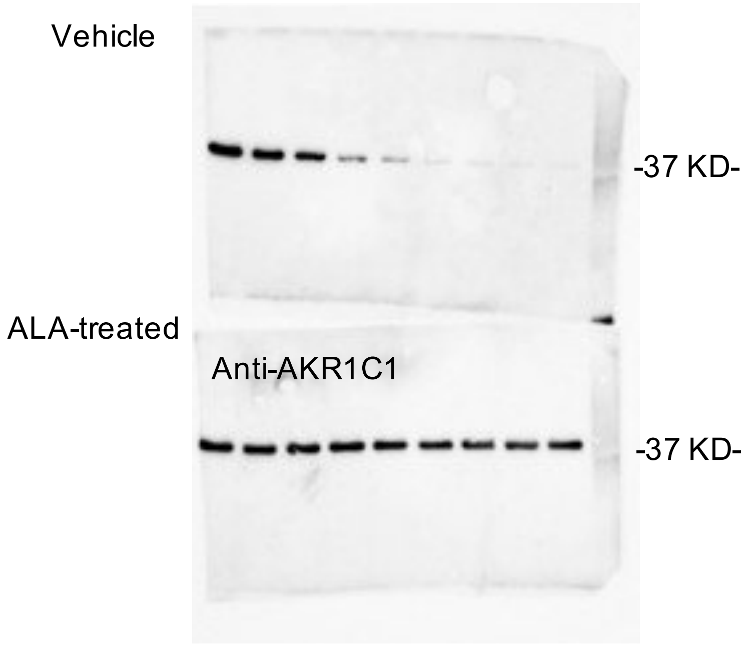


For exposure of GAPDH, blots were cropped and images were captured simultaneously.

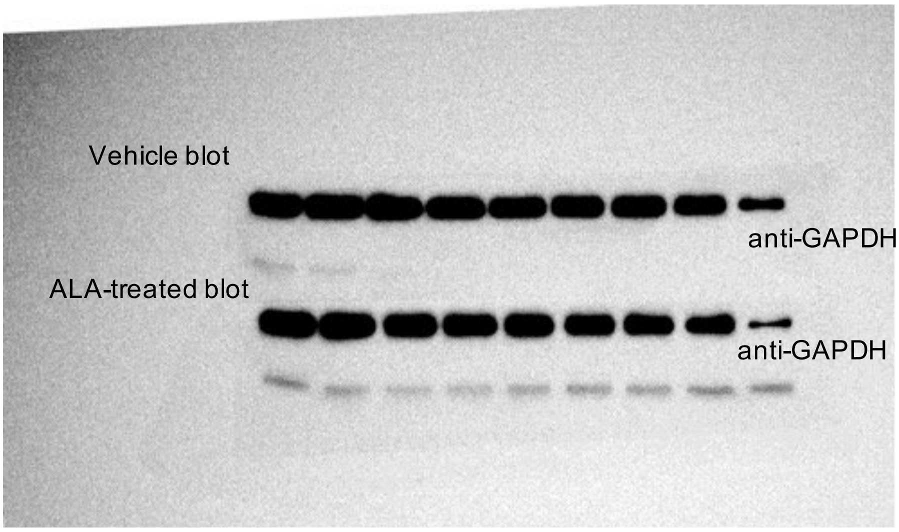

**S2. ITDRF experiment**

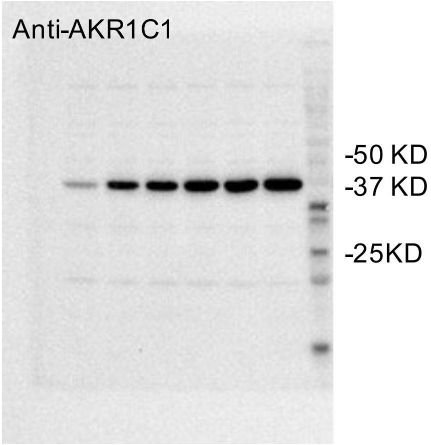


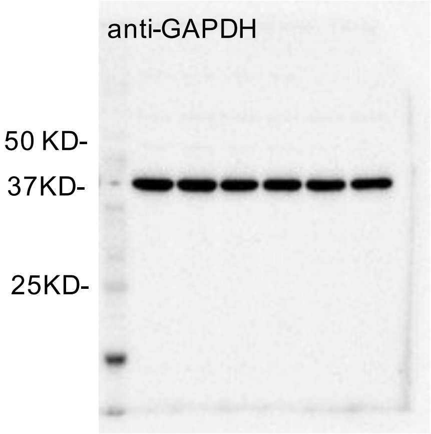

S3. STAT3 experiment-time dependent (Fig. 4g)

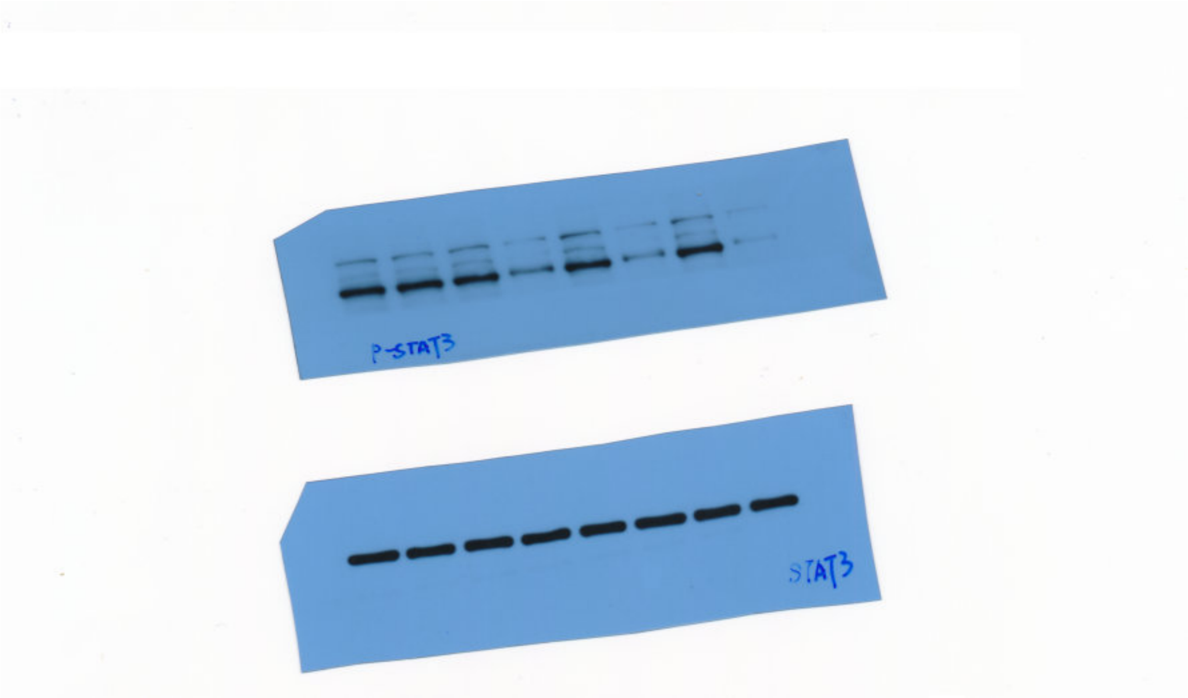


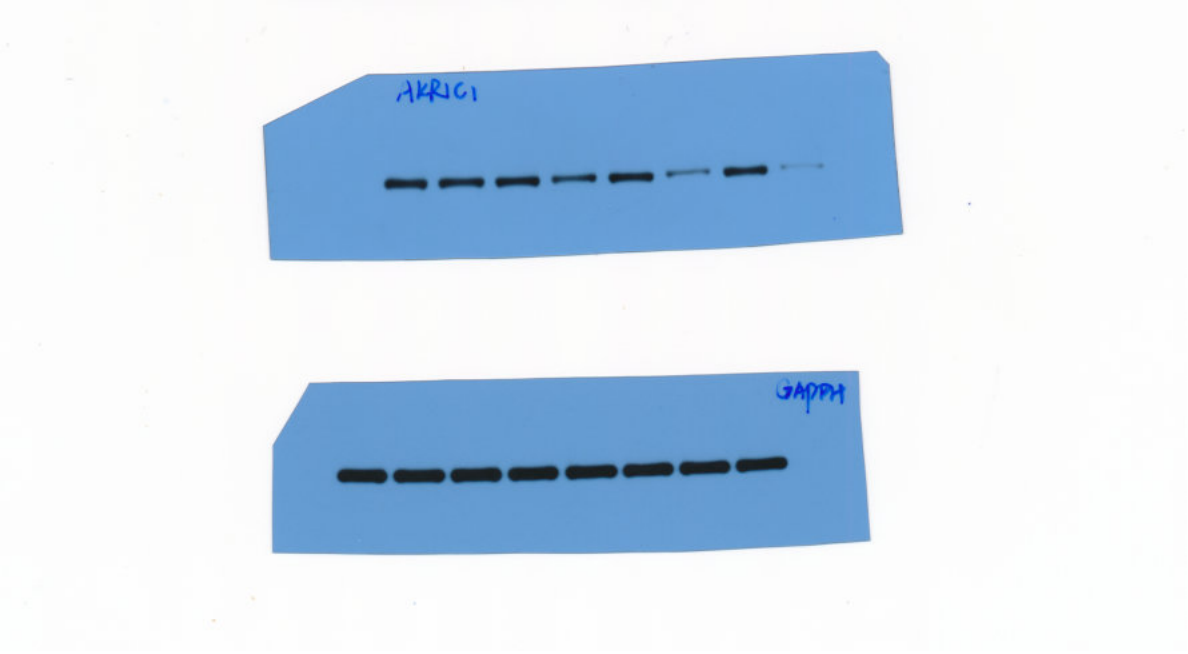

S4. STAT3 experiment-dose dependent (Fig. 4f)

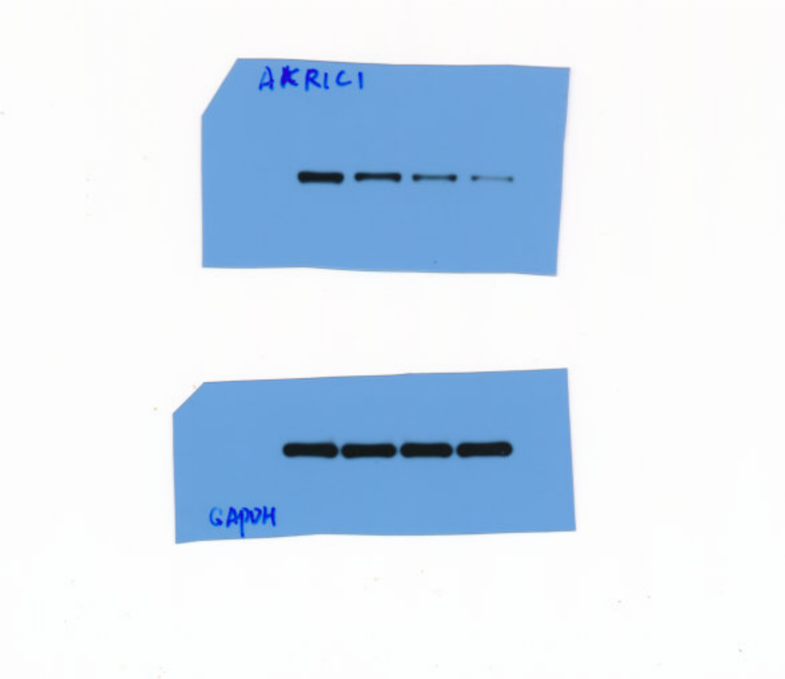


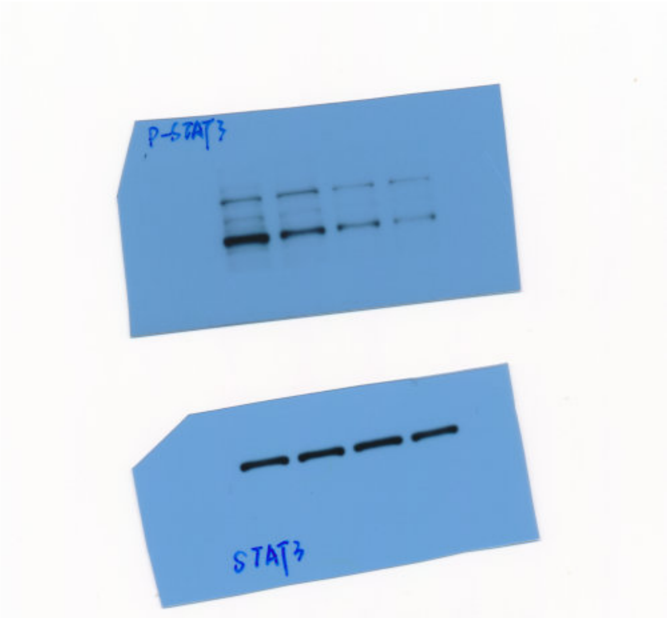

S5. STAT3 animal experiment (Fig.5f)

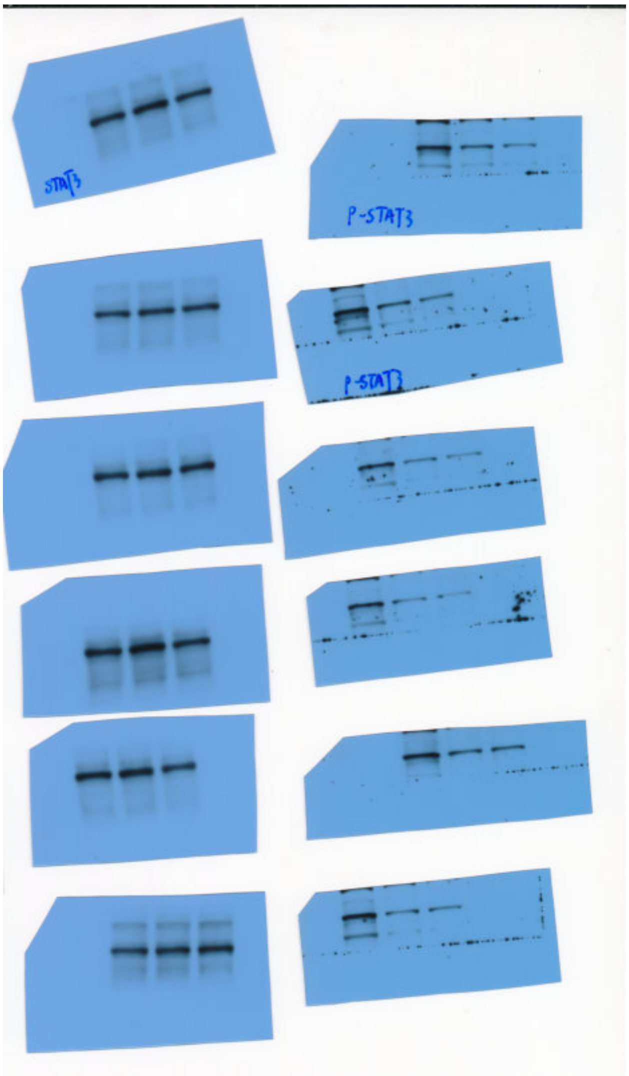


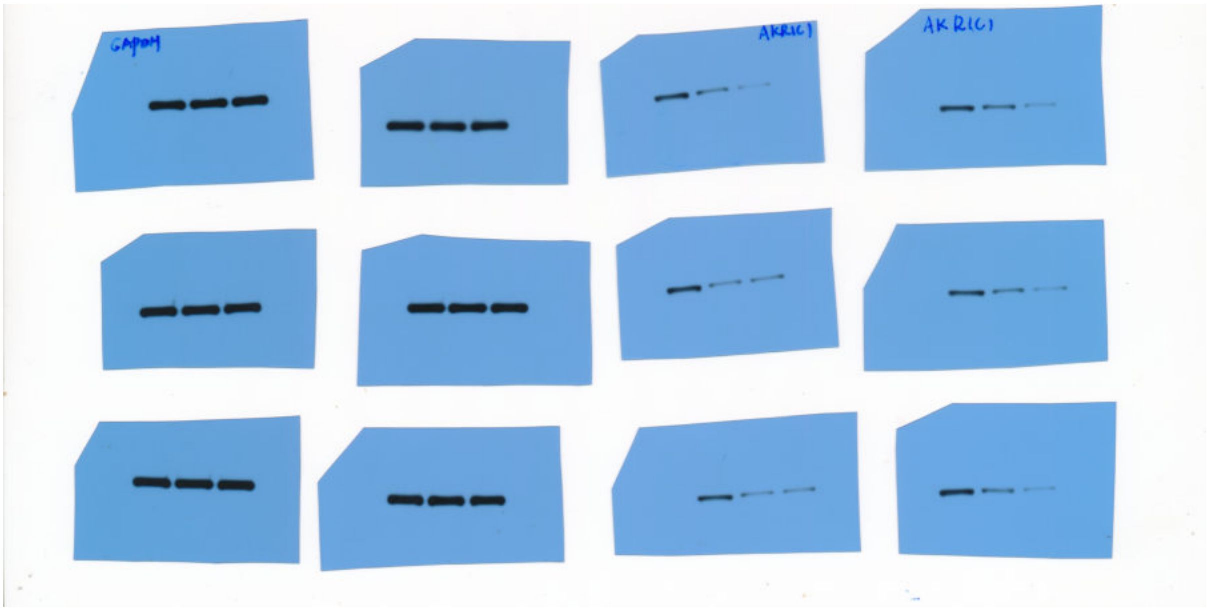

Supplement: Supplementary file 2 [file DataSheet1.PDF]
